# Progressive Thoracolumbar Tuberculosis in a Young Male: Diagnostic, Therapeutic, and Surgical Insights

**DOI:** 10.3390/idr16050080

**Published:** 2024-10-12

**Authors:** Dana-Georgiana Nedelea, Diana Elena Vulpe, George Viscopoleanu, Alexandru Constantin Radulescu, Alexandra Ana Mihailescu, Sebastian Gradinaru, Mihnea Orghidan, Cristian Scheau, Romica Cergan, Serban Dragosloveanu

**Affiliations:** 1 Department of Orthopaedics, “Foisor” Clinical Hospital of Orthopaedics, Traumatology and Osteoarticular TB, 021382 Bucharest, Romania; 2 Department of Anesthesiology and Critical Care, “Foisor” Clinical Hospital of Orthopaedics, Traumatology and Osteoarticular TB, 021382 Bucharest, Romania; 3 Department of General Surgery, County Hospital Ilfov, 050474 Bucharest, Romania; 4 Department of Medical-Clinical Disciplines, General Surgery, Faculty of Medicine, Titu Maiorescu University of Medicine and Pharmacy, 031593 Bucharest, Romania; 5 Department of Pneumology and Thoracic Surgery, “Marius Nasta” Institute of Pneumology, 050159 Bucharest, Romania; 6 Department of Physiology, The “Carol Davila” University of Medicine and Pharmacy, 050474 Bucharest, Romania; 7 Department of Radiology and Medical Imaging, “Foisor” Clinical Hospital of Orthopaedics, Traumatology and Osteoarticular TB, 021382 Bucharest, Romania; 8 Department of Anatomy, The “Carol Davila” University of Medicine and Pharmacy, 050474 Bucharest, Romania; 9 Department of Orthopaedics and Traumatology, The “Carol Davila” University of Medicine and Pharmacy, 050474 Bucharest, Romania

**Keywords:** spinal tuberculosis, tuberculous spondylitis, surgical management, medical imaging, expandable cage reconstruction, multidisciplinary approach

## Abstract

Objective: We present the case of a 26-year-old male with severe spinal tuberculosis of the thoracolumbar region. The patient suffered from worsening back pain over five years, initially responding to over-the-counter analgesics. Despite being proposed surgery in 2019, the patient refused the intervention and subsequently experienced significant disease progression. Methods: Upon re-presentation in 2022, mild involvement of the T12-L1 vertebrae was recorded by imaging, leading to a percutaneous needle biopsy which confirmed tuberculosis. Despite undergoing anti-tuberculous therapy for one year, the follow-up in 2024 revealed extensive infection from T10 to S1, with large psoas abscesses and a pseudo-tumoral mass of the right thigh. The patient was ultimately submitted to a two-stage surgical intervention: anterior resection and reconstruction of T11-L1 with an expandable cage, followed by posterior stabilization from T8-S1. Results: Postoperative recovery was uneventful, with significant pain relief and no neurological deficits. The patient was discharged on a continued anti-tuberculous regimen and remains under close surveillance. Conclusions: This paper presents details on the challenges of diagnosis and management of severe spinal tuberculosis, with emphasis on the importance of timely intervention and multidisciplinary care.

## 1. Introduction

The World Health Organization states in the Global Tuberculosis (TB) Report 2023 that more than 10 million people are diagnosed with TB every year, with more cases being registered in developing countries [1]. Although extra-pulmonary involvement is far less frequent than pulmonary TB, with an incidence estimated at around 10–15% of all diagnosed cases, spinal tuberculosis is the most common form of extra-pulmonary TB [2,3]. The diagnosis of extra-pulmonary TB should always be in the mind of the physician, especially in high endemic regions and in patients with persistent musculoskeletal complaints [4].

Spinal TB is usually found in the thoracolumbar region, after dissemination from the primary source, either hematogenous (frequent) or lymphatic, and can occur with or without concomitant pulmonary infection [5]. While spinal TB has distinctive imaging features that may allow for a high degree of diagnostic confidence, the gold standard for the clinical diagnosis of TB is represented by cultures [6,7].

The infection causes vertebral destruction, which leads to spinal deformity and compression of the spinal cord with further neurologic impairment and paralysis; however, this can be prevented by a rapid and correct diagnosis followed by suitable and personalized treatment [8].

Surgery aims to remove the infection, decompress the spinal cord, and provide stabilization of the spine [9]. While defining spinal instability in TB-infected patients may be challenging, several criteria such as younger age, pain, important deformities, or the involvement of junctional areas have been proposed as determining factors [10].

## 2. Case Presentation

### 2.1. Brief Case History

A 26-year-old male patient presented with complaints of junctional thoracolumbar back pain for the past 5 years, which was mild to moderate, but gradually increasing in severity, with no history of trauma or other pathologies. There was no history of cough, chest pain, or other related respiratory tract symptoms, no other pathologies present, no relevant past medical history, and no other prior hospitalization or transfusion history. He was immunized with the Bacillus Calmette–Guerin (BCG) vaccine, with a specific mark present over his left arm. He was a non-smoker, non-diabetic, had normal blood pressure and no history of alcohol abuse, AIDS, or other immunosuppressant disease, or treatment.

The patient was first diagnosed with under-documented spine pathology in 2019, for which surgery was proposed in another unit, which the patient refused. He came to our hospital in 2022 with mild involvement of the T12-L1 vertebrae for which he was referred to a Magnetic Resonance Imaging (MRI) investigation. In January 2023, percutaneous needle biopsy under fluoroscopy was performed and tuberculosis infection was identified on both histopathological examination and microbiological culture; however, no Rifampin resistance test was performed. On routine blood tests, a complete blood picture revealed hypochromic, microcytic anemia with decreased Hb levels of 12.5 g/dL, as well as decreased HTC 39/%, MCV 80.1/fL, and MCH 25.6/pg. The total WBC count was 7.49 × 10^3^/mm^3^ with 39.1% lymphocytes. The Erythrocytes Sedimentation Rate (ESR) was 24 mm per hour, the C-Reactive Protein (CRP) was 2.3 mg/dL (normal range 0–0.3 mg/dL), and the Fibrinogen (FBG) level was 406 mg/dL (normal range 180–350 mg/dL).

At the previous presentation, the patient did not show any neurological or mechanical symptoms, and the decision to start anti-tuberculous medication was made. The infection was mainly at the T12-L1 level with minor involvement of the other lumbar levels and small psoas abscesses present. The patient started the anti-tuberculous 4/4 7/7 treatment with Rifampin 600 mg, Isoniazid 300 mg, Ethambutol 1600 mg, Pyrazinamide 2000 mg under strict supervision and was monitored by the local Tuberculosis Center in the proximity of his home. During treatment, the patient reported that his back pain decreased in intensity from a visual analog scale (VAS) of 7–8 to VAS 3, but after the treatment was disrupted, the back pain worsened rapidly. He did not report to our hospital for any post-biopsy follow-up and we were not contacted by the local center.

In March 2024, the patient presented to our clinic for a check-up with a recent MRI where severe progression of the disease was shown. The patient had extensive tuberculosis involvement of the thoracolumbar spine from T10 to S1 with extensive abscesses in the right and left psoas muscle and a large pseudo-tumoral mass on the anterior proximal right thigh. The patient reported he had taken up to 1 year of anti-tuberculous treatment, with a short pause of one month due to the severity of adverse effects caused by antibiotics, which he previously interrupted. At the second admission, the patient was afebrile and hemodynamically stable. The patient presented numbness in both legs when standing, and severe back pain during rest and movements, especially with twisting and bending, which was partially relieved with over-the-counter analgesics. He denied fever, chills, or weight loss. The patient’s VAS for thoracolumbar spine pain was as high as 7–8 points and the Oswestry disability index (ODI) stated the quality of life was 36 points. The neurological status was grade E on the American Spinal Injury Association (ASIA) scale [11].

### 2.2. Physical Examination

**Figure 1:**
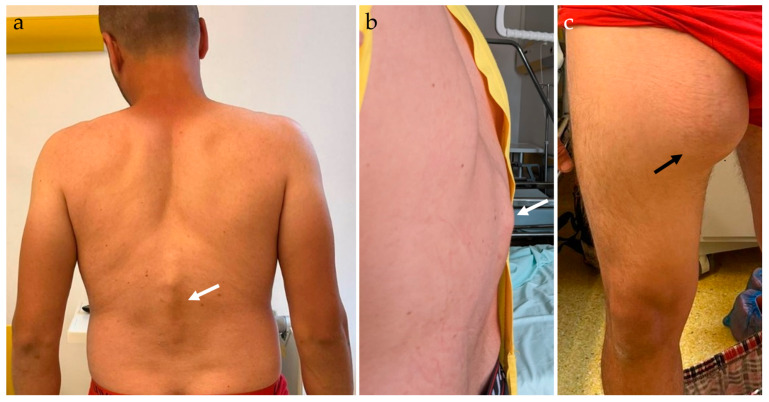
Clinical examination reveals thoracolumbar kyphosis deformity, with palpable gibbus (**a**,**b**, white arrow). Large pseudo-tumoral mass on the anterior and medial aspect of the right thigh, fluid-like consistency, representing an abscess (**c**, black arrow).

On general physical examination, the patient was anemic, with a body mass index (BMI) of 23.4, pulse of 63 bpm, and blood pressure (BP) of 134/71 mmHg. During the musculoskeletal system examination, thoracolumbar kyphosis was identified, with palpable gibbus, local tenderness, and local percussion pain in the spinous processes (Figure 1a,b). On neurologic examination, normal bulk, tone, and power in both lower limbs were reported, with intact and normal reflexes, and intact sensation. A massive pseudo-tumoral mass on the anterior and medial proximal right thigh was palpable, measuring approximately 20 × 20 cm, with fluid-like consistency, with no associated pain, intact sensation on the affected thigh, and normal-colored skin; the lesion grew quickly in the last couple of weeks and was suggestive of an abscess (Figure 1c).

Respiratory, cardiovascular, gastrointestinal, and renal system examination revealed no abnormal findings. Electrocardiogram and echocardiography were performed in order to assess cardiac function and pulmonary functional and imaging tests were performed to assess pulmonary status. The patient presented no other symptoms that would suggest multifocal bone disease.

### 2.3. Laboratory Investigations

Blood tests were performed on admission. On routine tests, the complete blood panel revealed hypochromic, microcytic anemia with decreased Hb levels of 12.9 g/dL, as well as normal HTC 40.9/%, decreased MCV 78.5/fL, MCHC 31.6/g/dL, and MCH 24.8/pg. The total WBC count was 7.40 × 10^3^/mm^3^ with 28.7% lymphocytes and 63.9% neutrophils. The ESR was 13 mm per hour; the FBG level was 347 mg/dL. Renal function examination showed urea 24 mg/dL, creatinine 0.72 mg/dL, and uric acid 4.7 mg/dL. Liver function examination showed aspartate aminotransferase 23 U/L, alanine aminotransferase 26 U/L, and albumin 2.3 g/dL.

### 2.4. Imaging Examinations

**Figure 2:**
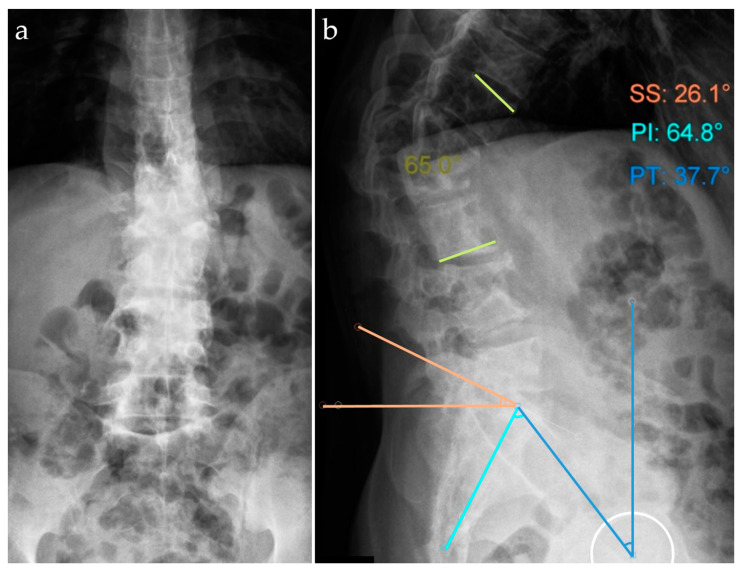
Preoperative X-ray examination, anteroposterior (**a**) and lateral (**b**) views of the thoracolumbar spine. The angle of kyphosis measured 65 degrees in the sagittal plane. Pelvic parameters: SS = sacral slope, PT = pelvic tilt, PI = pelvic incidence.

**Figure 3:**
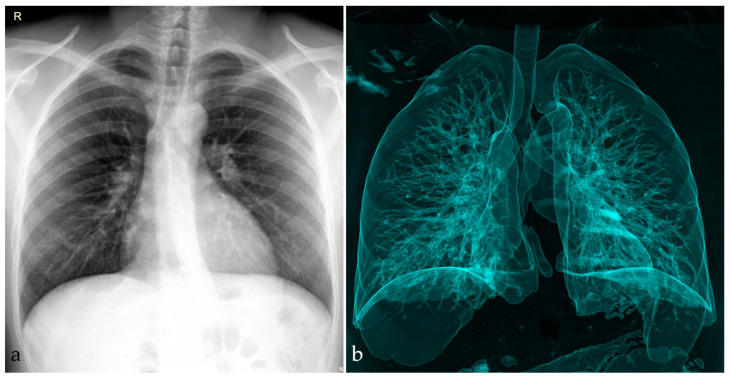
(**a**) Chest X-ray in postero-anterior projection showing no pulmonary lesions suggestive of lung tuberculosis; (**b**) virtual rendering of the airways showing no cavitary lesions, bronchiectasis, or other airway lesions (made with RadiAnt DICOM Viewer (Medixant, Poznań, Poland), https://www.radiantviewer.com (accessed on 19 July 2024)).

A radiographic examination was performed, with anteroposterior (AP) and lateral (L) views of the thoracolumbar spine in a standing position. The spine radiographic examination showed massive destruction of T12 and L1 vertebral bodies. The kyphosis angle was measured at 65 degrees in the sagittal plane, along with the pelvic parameters as follows: sacral slope = 26.1 degrees, pelvic tilt = 37.8 degrees, pelvic incidence = 64.8 degrees (Figure 2) [12,13]. On routine imaging, no important abnormalities in the lungs were identified (Figure 3).

**Figure 4:**
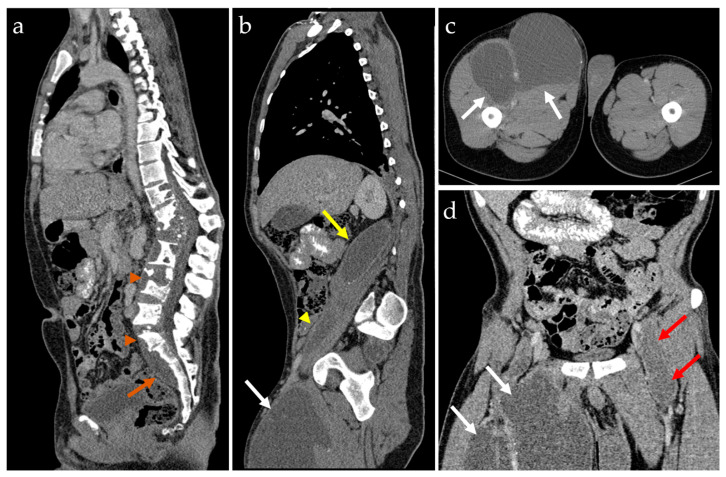
Contrast-enhanced computed tomography of the thorax, abdomen, and pelvis. Sagittal-reformatted images (**a**) showing fused abscesses along the anterior spine (orange arrowheads) leading to a presacral abscess (orange arrow); sagittal-oblique views (**b**) show a paravertebral abscess (yellow arrow) fused alongside the right psoas muscle (yellow arrowhead) leading to the right inguinal multiloculate parafluid collection (white arrows), also detailed in the transverse plane (**c**) and coronal (**d**) reformat. A left-side parafluid collection of smaller size is seen in the left inguinal area, emerging from the pelvis on the surface of the iliopsoas muscle (red arrows).

Computed tomography (CT) with contrast media was performed in order to assess the extent of the lesions and to perform preoperative planning (Figure 4). As the patient had no other symptoms, we did not consider evaluation for multifocal bone disease.

**Figure 5:**
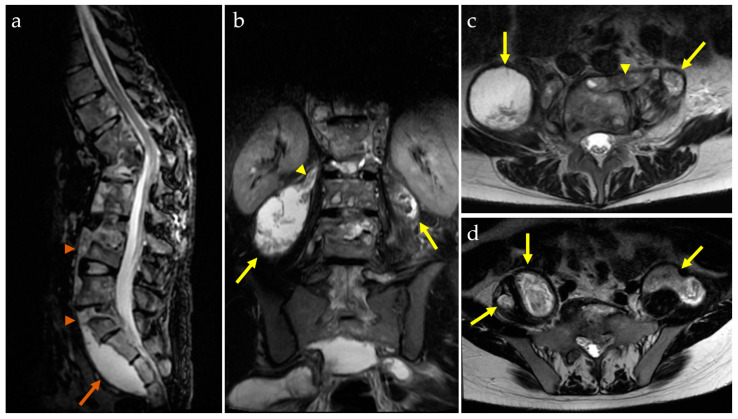
MRI of the spine: sagittal STIR image (**a**) reveals a prevertebral abscess (orange arrowheads) extended to the presacral region (orange arrow) associated with the destruction of mainly T12 and L1 vertebral bodies, but with no apparent involvement of the spinal cord; coronal STIR image (**b**) shows bilateral massive paravertebral abscesses (yellow arrows) communicating with the intervertebral space on the right side (yellow arrowhead); transverse T2-WI (**c**,**d**) reveals destruction of the L1 vertebral body, with multiple encapsulated abscesses on both sides of the spine (yellow arrows) in communication with the intervertebral space on the left side (yellow arrowhead).

An MRI with screening of the whole spine was performed in order to detect potential infra-clinical lesions undetectable by CT and to evaluate the spinal cord status. The MRI revealed signal anomalies in the T12, L1, and L2 vertebral bodies and posterior elements, with destructive processes involving T12, L1, and L2 vertebral bodies, along with large paravertebral lobulated soft-tissue collections, emerging from T11-T12 and extending into the anterior and posterior epidural space, then fused into the bilateral iliopsoas compartment (Figure 5).

### 2.5. Management and Surgical Procedures

The decision to operate was made and the patient agreed to undergo surgery. Because of the severity of the infectious involvement and the complexity of the required surgery, a multidisciplinary team of doctors was assembled to manage this case. During the board meeting, the surgical approach options as well as options for reconstructing the spine were reviewed.

**Figure 6:**
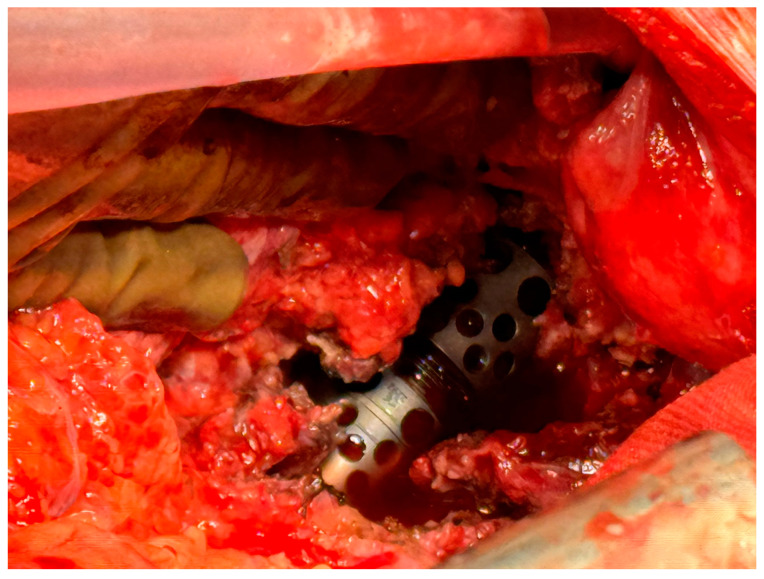
Intra-operative picture of the anterior aspect of the expandable cage used for reconstruction of the T11, T12, and L1 vertebral bodies that were resected. The major vessels are retracted to the left.

After successful general anesthesia, with central venous catheter and artery access, the patient was placed in a right lateral decubitus, with routine skin disinfection and towel laying. With the help of a thoracic surgeon and a general surgeon, a left-sided retroperitoneal approach was performed, with the skin incision centered over the 10th rib, with detachment of the left crus and access to the thoracic cavity in order to expose the T11, T12, and L1 vertebral bodies. The left-sided approach was chosen because of the fewer risks presented by the mobilization of the aorta rather than the vena cava on the opposite side. After the ligature of the segmental vertebral vessels, the resection of the T11, T12, and L1 vertebral bodies was performed and an expandable cage filled with bone autograft mixed with vancomycin was positioned (Figure 6). The pathological material evacuated from the psoas abscesses and the thoracic vertebral bodies involved was sampled for histopathological examination and Giemsa and Ziehl–Nelson stains and cultures for aerobic and anaerobic bacteria and fungi. Genetic and biological testing for tuberculosis were also performed. After collecting the samples for the culture tests, the patient was administered intravenous antibiotics. After the wound was thoroughly rinsed with normal saline and no active bleeding was detected, a pleural drain was used and the incision was sutured layer by layer.

The patient was then carefully placed in a prone position, routine skin disinfection was conducted, and towel laying was performed. A posterior median longitudinal incision was made, centered over the thoracolumbar spinous processes from T8 to L5. The skin, the subcutaneous layer, and the fascia were cut layer by layer. After detachment of the paraspinal muscles subperiosteally, a retractor was placed in order to preserve the operating field. Transpedicular stabilization was performed with poly-axial pedicle screws connected by rods, with posterior stabilization from T8 to S1. The L3-L4 and L5-S1 abscesses were drained from posterior through the right L3 pedicle and the L5-S1 disc space, with debridement of any macroscopically modified tissue, and the pathological tissue was sampled for histopathological examination and to perform Giemsa and Ziehl–Nelson stains and cultures for aerobic and anaerobic bacteria and fungi. Genetic and biological testing for tuberculosis was also performed. After the wound was thoroughly rinsed with normal saline and no active bleeding was detected, vancomycin powder was applied, a vacuum drain was inserted, and the incision was sutured layer by layer. The right psoas and thigh abscess were drained through a retrograde tube at the end of the surgery.

**Figure 7:**
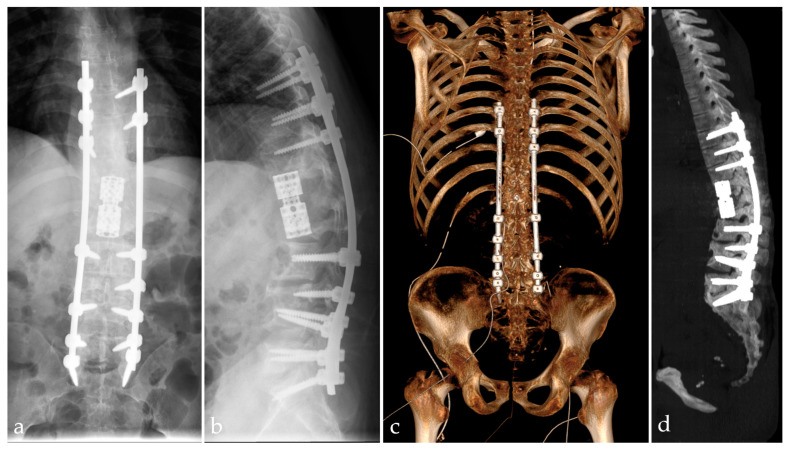
AP (**a**) and lateral (**b**) X-ray views of the thoracolumbar spine, with T11, T12, and L1 vertebral body resection and reconstruction with expandable cage, along with posterior fixation from T8 to S1 with pedicle screws and rods. Post-operative CT with virtual rendering (**c**) and maximum intensity projection (**d**) for the assessment of the correct placement of the hardware. VRT made with RadiAnt DICOM Viewer (Medixant, Poznań, Poland), https://www.radiantviewer.com (accessed on 19 July 2024).

The patient was extubated in the following hour after the end of the surgery, monitored in the intensive care unit for 48 h, and then stepped down to the common ward. Standard AP and lateral views of the post-operative thoracolumbar spine as well as a CT examination of the spine in order to assess the correct positioning of all implants were performed (Figure 7).

### 2.6. Postoperative Management and Recovery

**Figure 8:**
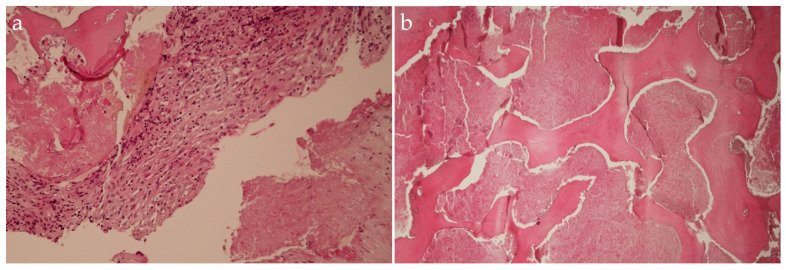
Histopathological examination in hematoxylin-eosin staining, ×10 (**a**,**b**), shows fibro-connective tissue with chronic inflammatory cells, caseous necrosis, and fragments of destructed bone trabecula, suggesting the diagnosis of TB spondylodiscitis.

The antibiotic therapy with Linezolid 1200 mg and Levofloxacin 750 mg was continued until the results from histopathological examination, stains, and cultures were ready. The histopathological examination showed fibro-connective tissue with chronic inflammatory cells, caseous necrosis and fragments of destructed bone trabecula, suggesting the diagnosis of TB spondylodiscitis (Figure 8).

A molecular GeneXpert test was also performed in order to identify a potential gene for Rifampin resistance, which came back positive for tuberculosis infection and negative for any resistance. The patient was then put on standard anti-tuberculous treatment as the assumption was that he was not adherent to the initial treatment.

The patient was stable, with no motor or sensory deficits after surgery. At 48 h after surgery, he began mobilization with a thoracolumbar Hessing Brace and the help of a rehabilitation and physical therapy specialist. Following surgery, the patient experienced a decrease in the intensity of the thoracolumbar pain, and was able to walk without the use of any assistive device. The patient remained afebrile and the wound healed without complications. During the hospital stay, post-operative inflammation levels showed a decreasing trend and the patient showed improvement in his clinical condition. He was discharged 20 days after surgery, with a good clinical status, afebrile, hemodynamically stable, and able to walk without the need for an assistive device. He was further referred to the Institute of Tuberculosis and Lung Diseases for further continuation of the anti-tuberculous treatment.

At the 6-week follow-up, complete blood count, C-reactive protein, ESR, FBG and liver, and renal function were assessed. The patient showed hypochromic, microcytic anemia with decreased Hb levels of 9.7 g/dL, as well as decreased HTC 32.3/%, MCV 81.6/fL, MCHC 30.2/g/dL, and MCH 24.6/pg and total WBC count of 5.07 × 10^3^/mm^3^ with 38.5% lymphocytes and 48.8% neutrophils. The ESR was 22 mm per hour and the FBG level was 384 mg/dL. Renal function examination showed urea 31 mg/dL, creatinine 0.69 mg/dL, and uric acid 4 mg/dL. Liver function examination showed aspartate aminotransferase 25 U/L and alanine aminotransferase 44 U/L, and the C-reactive protein was 2.6 mg/dL. Standard anteroposterior and lateral views of the thoracolumbar spine were performed. The patient was afebrile, with a good clinical status, walked without the use of any assistive device, with a thoracolumbar Hessing Brace, had mild thoracolumbar pain (VAS 2-3/10), and continues anti-tuberculous treatment under strict supervision.

## 3. Discussions

Tuberculosis is still a high-prevalence infectious disease in under-developed and poor countries, being a huge burden for the health and social economy [14]. Spinal tuberculosis is the most frequent extra-pulmonary form and can have various presentation forms, from simple and benign back pain to severe cases of rapidly installed paraplegia [15]. The most frequently affected area is the lower thoracic spine, followed by the upper lumbar spine [16]. It is usually more frequent among the male population and people with comorbidities, immunosuppression, and older age [17]. In contrast, our patient was a young and active patient, without any known comorbidities or immunosuppression, who had neither pulmonary TB symptoms nor any pulmonary lesions, and had no known contact with a patient with active TB.

Patients with mild symptoms without neurologic impairment are treated with conventional medical treatment [18]. This was also the case for our patient, who first received anti-tuberculous treatment as his only complaint was back pain without any neurologic deficit. After one year of conventional medical treatment and shortly after discontinuation of oral antibiotics, the patient presented with aggravated back pain, along with the formation of cold abscesses. Indications for surgical treatment in such cases could be summed up as follows: important vertebral destruction with kyphosis or large paraspinal abscesses, spinal instability, severe back pain, with or without radiculopathy, or other neurologic impairment [19]. Conversely, surgical treatment is recommended for patients with spinal cord compression or severe deformities. The most appropriate surgical approach will be chosen after thoroughly assessing the patient and their disease. The surgical approach may vary on the affected area or the symptoms, as a patient with severe pain may benefit from a posterior stabilization only, whereas a patient with spinal cord compression is a candidate for spinal decompression [20]. As was the case of our patient, spinal TB involving the thoracolumbar region is a challenge from the biomechanical point of view [21]. In our patient, because of the severe deformity present, a combined anterior and posterior approach was considered beneficial.

Three-dimensional printing enhances surgery for complex spinal deformities and advanced spinal diseases, like infections and tumors, by providing precise anatomical models for better preoperative planning [22,23]. It enables the creation of patient-specific surgical guides, improving implant-placement accuracy and reducing operative time [24,25]. Additionally, 3D-printed personalized devices and instruments tailored to individual anatomy contribute to better surgical outcomes and fewer complications [26,27]. Digital 2D or 3D preoperative planning is essential for assessing the type and size of required implants as well as for fitting 3D-printed elements [28,29,30].

There are different opinions regarding the anti-tuberculous treatment duration, between 6 and 12 months of medical therapy, with constant monitorization of the toxicity of anti-tuberculous drugs, with a preference for prolonged medical therapy time of 12 months [31]. A multidisciplinary team, including pulmonary, infectious diseases, and a laboratory specialist, is needed in order to establish the correct medical anti-tuberculous therapy [32,33].

After surgery, protection of the spine construct and cord is vital; therefore, our patient was advised to wear the thoracolumbar Hessing Brace 24/24 h, for at least 6 weeks [34].

Implant failure is a potential complication in all types of stabilization and prosthetic surgery [35,36,37]. Fusion-related complications are also a possibility, as well as systemic adverse effects directly related to the disease and magnitude of the intervention [38,39]. A thorough knowledge of potential complications and adequate postoperative monitoring are essential in improving patient outcomes.

## 4. Conclusions

Spinal tuberculosis should be diagnosed as early as possible with imaging, laboratory, and histopathological examinations and promptly treated in order to prevent deformities and irreversible destruction of the spinal column [40]. The diagnosis of extra-pulmonary TB should always be in the mind of the physician, especially in high-endemic regions [41]. After a positive diagnosis, depending on the severity of the disease, medical-only or medical and surgical treatment will be applied. The current duration of treatment is prolonged from 6 to 12 months, and sometimes even 18 months, with imaging monitoring of the therapeutic effects every 6 months. Testing for gene resistance to Rifampin should be performed from the first attempt of treatment [42]. The complexity of this disease and its many faces will often require personalized and multidisciplinary patient treatment.

## Data Availability

The data presented in this study are available on reasonable request from the corresponding author.
